# Evaluation of oxidant/antioxidant status, metabolic profile and milk production in cows with metritis

**DOI:** 10.1186/s13620-020-00161-3

**Published:** 2020-05-27

**Authors:** Karolína Mikulková, Romana Kadek, Jaroslav Filípek, Josef Illek

**Affiliations:** Large Animal Clinical Laboratory, Faculty of Veterinary Medicine, University of Veterinary and Pharmaceutical Sciences, Palackého tř. 1946/1, 612 42 Brno, Czech Republic

**Keywords:** Oxidative stress, Metritis, Lipid peroxidation, Antioxidants, Dairy cows

## Abstract

**Background:**

The aim of the study was to evaluate oxidant/antioxidant status in 21 Holstein dairy cows with metritis compared to 8 healthy controls. Blood samples were taken during the first 21 days postpartum. Malondialdehyde (MDA), a marker of oxidative stress, total antioxidant status (TAS) and antioxidant parameters such as glutathione peroxidase (GPx), selenium (Se), vitamins A and E and beta-carotene were determined from all cows. The differences in beta-hydroxybutyrate (BHB), non-esterified fatty acids (NEFA), calcium, bilirubin concentrations and aspartate aminotransferase (AST) activity were also monitored, as were milk production and milk composition. Metritis was defined by an unpleasant discharge of varying color (milky-grey/brown/sanguineous) and consistency (muco-purulent/purulent/watery) and by the presence of increased temperature (> 38.5 °C) in cows within 21 days postpartum. Rectal examination revealed increased uterine size, thickened uterine wall and increased uterine tone. The affected cows had significantly reduced daily milk production. Additionally, hematological parameters and haptoglobin concentration were also measured in metritic cows.

**Results:**

Higher MDA concentration (*P* <  0.001) was recorded in cows with metritis, while vitamin A and vitamin E concentrations were lower (*P* <  0.01) compared to healthy cows. Higher BHB (*P* <  0.05), NEFA (*P* <  0.05), AST (*P* <  0.05) and bilirubin (*P* <  0.001) concentrations was recorded in cows with metritis as compared to the control group. Significant differences in beta-carotene concentration, GPx activity, and Se, TAS and Ca concentrations in cows with metritis compared to control group were not observed in the present study (*P* >  0.05). Milk production was decreased in the cows with metritis (*P* <  0.001) and alterations in milk composition were also observed in metritic cows as compared to healthy cows.

**Conclusions:**

The results of the study showed that cows with metritis in early postpartum are exposed to a higher degree of oxidative stress and that the incidence of metritis can negatively affect milk production in dairy cows.

## Background

The periparturient period is considered critical for high yielding cows (more than 25 l/day), particularly when it comes to metabolism, immunity, health, fertility, milk production and hormonal changes [1, 2]. The immune system of high yielding cows is depressed due to peripartal stress during this period. Periparturient stress is caused predominantly by hormonal and metabolic changes (negative energy balance), and a lack of the vitamins, minerals and antioxidants that are associated with the requirements of the growing fetus and the onset of lactation [1, 3, 4].

The lumen of the uterus after calving is often contaminated with bacteria that may cause disease that can also lead to infertility. The immune response of postpartum cows and the number and type of bacteria are important factors in the development of uterine disease [5]. Polymorphonuclear neutrophils, which are attracted by chemokines such as interleukin 8, play a key role in the immune response. However, uterine involution and endometrial regeneration after parturition are also important mechanisms for eliminating bacterial contamination from the uterus [6]. Uterine involution after calving is the complex process that also includes uterine contractions, physical shrinking, necrosis, sloughing of caruncular material and regeneration of the endometrium [7]. Although the uterus of postpartum cows can be contaminated with a variety of bacteria, this may not necessarily be associated with clinical disease. Most cows eliminate these bacteria from the uterus within a few weeks. The pathophysiology for development of uterine infection includes adhesion, colonization / penetration of pathogenic microorganisms into the epithelium of the mucosa, and / or the release of bacterial toxins. Therefore, uterine contamination by bacteria (common in all cows after parturition) and uterine infection (developed uterine disease with specific clinical signs) should be differentiated [8]. Furthermore, it appears that uterine infections are also associated with endocrine factors, especially progesterone, which suppress the immune defenses of the uterus. The immunosuppressive effect of progesterone from the prolonged corpus luteum contribute to the progression of uterine contamination into uterine infection [9, 10]. Therefore, therapy with exogenous prostaglandin F_2_ alpha or an intrauterine antimicrobial is effective [11]. When the infection is limited to the endometrium, inflammation does not extend deeper than the stratum spongiosum of the uterus [12, 13]. On the other hand, in the case of metritis, all layers of the uterus wall exhibit inflammation, edema, leukocyte infiltration and myometric degeneration [9]. *Escherichia coli, Trueperella pyogenes, Fusobacterium necrophorum, Prevotella melaninogenica, Streptococcus pyogenes, Bacteroides spp., Pseudomonas spp. and Staphylococcus spp.* are common pathogens in endometritis and metritis [14, 15]. Up to 40% of dairy cows are exposed to uterine disease within a week of parturition. Metritis is present in about 20% of cows within 21 days postpartum. Endometritis occurs in 15–20% of all postpartum cows because of persistent bacterial growth for 3 weeks or more postpartum [11, 16].

Clinical signs of metritis are well known. Infection usually occurs within the first 21 days (most often 10 days) after calving. Malodorous discharge, from reddish brown watery discharge to more viscous lighter discharge, from the uterus is an essential clinical sign of metritis. Fever (> 39.5 °C) is a frequent accompanying symptom. Other clinical signs such as apathy, decreased milk yield, dehydration, anorexia, increased heart rate and dehydration can be also observed in the metritis. An enlarged uterus can be detected by rectal examination. Metritis often occurs in association with a retained placenta, the birth of twins, dystocia and premature birth [9, 11].

Oxidative stress in the periparturient period is known to be one of the factors contributing to increased susceptibility to diseases like mastitis, metritis, mammary edema and retained fetal membranes [17, 18]. Many studies have been carried out to support this fact [19–21]. Metabolic requirements associated with late pregnancy, calving and the onset of lactation contribute to increased production of reactive oxygen species (ROS) [22] which are known to cause lipid peroxidation followed by oxidative stress and damage tissue. Studies show that the cow can usually manage free radical production with sufficient antioxidant reserves, but oxidative stress may occur in the event of an imbalance between ROS production and the availability of antioxidant molecules [17, 23]. The results of the study by Kizil et al. [24] indicated that the antioxidant system was weakened and peroxidation reactions were accelerated in cows with metritis.

In relation to current literature, we have attempted to provide a comprehensive overview / relationship in the diagnosis of metritis by clinical and laboratory examination, oxidative stress using oxidative and antioxidant markers, the basic metabolic profile characteristic of postpartum period in cows and milk production and its composition during the first 30 days of lactation. The main purpose of the study was to monitor the relationship between oxidative stress and the occurrence of metritis postpartum in dairy cows. Therefore, the objective of this study was to compare the marker of oxidative stress and antioxidant status – malondialdehyde (MDA), total antioxidant status (TAS), glutathione peroxidase (GPx), selenium (Se), vitamins A and E, and beta carotene, and additionally the metabolic profile – beta-hydroxybutyrate (BHB), non-esterified fatty acids (NEFA), calcium (Ca), aspartate aminotransferase (AST) and bilirubin between cows with metritis and a healthy control group. Based on the above findings, we expect an increase in oxidative stress indicators and a decrease in antioxidants in metritic cows with compared to the control group. Another objective was to assess whether metritis affects the milk production of dairy cows. Milk production was recorded and also milk composition including milk fat, protein, lactose, urea and somatic cell count (SCC) were determined in cows of both groups.

## Materials and methods

### Animals

The study was carried out using 21 Holstein dairy cows with metritis (M group) and 8 healthy controls (CO group), with a mean milk production of 10,249 l, at a farm located in the village of Uherčice (Břeclav, South Moravia, Czech Republic). The farm was chosen because of the incidence of metritis in the cows and also because of the good cooperation with the staff. The experiment was carried out over 5 months (april-august 2018). Cows in the control group had no complications of the diseases, none were treated for diseases during the experimental period, and none of them had calving complications (only single pregnancy). Cows in M group were selected on the basis of clinical signs of disease (listed below). Cows (ranging) from the first to the sixth lactation were included in the experiment, specifically the parity of CO group was 1–4 (average 1.75) and M group 1–6 (average 2.29). There were no instances of death or culling. All of the cows were fed a total mixed ration (TMR) according to the antepartum and the postpartum period (Table 1).

**Table 1:** Total mixed ration composition (kg/day/cow)

Ingredients	*ante partum*	post partum
Alfalfa hay	2	1
Barley straw	2.2	0
Concentrate^a^ (DOVP)	0	6
Concentrate^b^ (DOVP – a.p.)	2.8	0
Post-extraction repessed meal	0.8	0
Pamitate	0	0.15
MP iont –^c^	0.5	0
High moisture corn	0	3
Brewers grains	0	4
Alfalfa haylage	0	6
Maize silage	15	19

^a^DOVP – complementary feed for lactating dairy cows; ^b^DOVP – a.p. - complementary feed for dairy cows *ante partum;*^c^MP iont - – mixture of anions, mineral suplements and protein concentrate (to prevent postpartum hypocalcaemia)

### Study design

The cows were monitored for metritis until 21 days after parturition. Metritis was detected by clinical and rectal examination performed once (always by the same person). Metritis was defined by an unpleasant discharge of varying color (milky-grey/brown/sanguineous) and consistency (muco-purulent/purulent/watery) and then by the presence of increased temperature (> 38.5 °C). Rectal temperature and discharge character, color and odor were observed (Table 2). Calving difficulty and the incidence of other diseases were also recorded. Blood samples were taken for laboratory examination and also body condition score (BCS) was recorded. In case of control group, only blood samples were taken during the first 21 days after parturition and BCS as in metritic cows was recorded - postpartum at blood collection of the cows and also 1 week before expected calving (CO group – 3.38 ± 0.26 p.p., 3.91 ± 0.40 a.p.; M group – 3.08 ± 0.16 p.p., 3.92 ± 0.25 a.p.).

**Table 2:** Clinical signs in cows diagnosed with metritis

Cow ID	DIM^a^	Rectal temperature (°C)	Character of discharge	Discharge color	Discharge odor	Calving difficulty^b^	Note
230,388	7	39.4	purulent	brownish	yes	2	
205,602	14	39.4	muco-purulent	milky grey	yes	1	
359,985	7	39.2	muco-purulent containing flecks	yellowish	yes, mild	1	stillbirth (6 days prior to expected calving)
256,949	11	39.0	purulent	milky grey	yes	3	+ retained placenta^c^
217,991	10	38.4	muco-purulent	milky grey	yes, mild	1	
217,982	11	39.0	muco-purulent	milky grey, sanguineous	yes	1	
230,390	6	39.3	watery, large amount	brown	yes	3	
205,626	5	39.3	purulent	Brown	yes	2	+ retained placenta^c^
217,973	5	39.2	purulent	Brown	yes	1	+ retained placenta^c^
226,235	6	38.9	watery	Brown	yes	1	
243,791	11	38.9	muco-purulent	milky grey, sanguineous	yes	1	
256,982	9	39.2	muco-purulent	milky grey, sanguineous	yes	1	
257,007	8	38.7	watery	brown, sanguineous	yes	1	
230,446	1	39.6	muco-purulent	brown, sanguineous	yes	1	
238,907	15	38.4	muco-purulent/ watery	milky grey, sanguineous	yes	1	
257,050	9	39.0	purulent	brown, sanguineous	yes	2	
196,466	7	39.6	muco- purulent	yellow- brown	yes	1	
256,948	8	39.0	purulent	milky grey, sanguineous	yes, mild	3	
243,779	12	39.6	purulent	milky grey	yes, mild	1	
256,971	4	39.6	muco-purulent, flocks	milky grey, sanguineous	yes, mild	1	
195,934	12	39.0	muco-purulent, flocks	milky grey, sanguineous	yes, mild	1	

^a^Days in milk; ^b^Calving difficulty: 1 – spontaneous parturition, no assistance; 2 – assistance by one person without the use of mechanical traction; 3 – assistance by 2 or more people/veterinarian/assistance with mechanical traction; ^c^non-expelled placenta more than 24 h after delivery

Indicators of oxidative stress, antioxidant status and metabolic profile were determined in a total of 29 blood samples. In cows with metritis, hematological parameters and haptoglobin concentration were also determined as an additional examination of health status. Individual milk yield in the first month of lactation was electronically recorded. Milk composition, including milk fat, protein, lactose, SCC and urea, was analyzed at the Milk Testing Laboratory, ČMSCH a.s., Brno, Czech Republic.

### Sampling and analysis

Blood samples were collected from the tail into Hemos sampling tubes (HEMOS H-02, GAMA Group, Czech Republic) without anticoagulant for serum determination (TAS, vitamins A and E, beta carotene, BHB, NEFA, Ca, AST, bilirubin, haptoglobin) and into Hemos sampling tubes (HEMOS H-02, GAMA Group, Czech Republic) with heparin anticoagulant for determination of whole blood (GPx, Se) and plasma (MDA). Blood samples for determination of hematological parameters (WBC, lymphocytes, monocytes, granulocytes, RBC) were only collected from metritic cows into sampling tubes with EDTA (ethylenediaminetetraacetic acid) anticoagulant. For serum samples, blood was allowed to clot and kept at room temperature until the separation of serum. Clotted blood was centrifuged at 3000 rpm for 10 min. Serum samples were either immediately used for analysis or stored at − 70 °C. For plasma samples, whole blood was centrifuged at 3000 rpm for 10 min. Plasma samples were stored at − 70 °C until analysis. Blood samples for hematological determination were analyzed immediately after collection.

### Hematological parameters and haptoglobin concentration

Hematological parameters such as WBC, lymphocytes, monocytes, granulocytes and RBC were measured using an automatic BC–2800 Vet hematology analyzer (Mindray, China). Haptoglobin concentration was analyzed with a Haptoglobin kit (Tridelta Development Ltd., Ireland) using a colorimetric assay by an automatic Konelab 20XT biochemical analyzer (Thermo Fisher Scientific, Finland).

### Oxidant/antioxidant status

The plasma MDA concentration was determined using the high-performance liquid chromatography (HPLC) system Ultimate 3000 (Dionex, USA), after prior derivatization of MDA with 2, 4-dinitrophenylhydrazine as described previously [25]. The selenium concentration in whole blood was analyzed using hydride generation atomic absorption spectrometry – HG AAS (SOLAAR, Thermo Scientific, USA). The samples for Se determination were prepared by mineralization with HNO_3_ and H_2_O_2_ using the microwave digestion system ETHOS TOUCH CONTROL (Milestone, Italy) followed by evaporation. Whole blood GPx activity was measured with a RANSEL kit (Randox Laboratories Ltd., UK) using a UV method based on that of Paglia and Valentine [26]. Serum TAS was also measured using standardized kits supplied by Randox Laboratories Ltd. For determination of GPx and TAS an automatic Konelab 20XT biochemical analyzer (Thermo Fisher Scientific, Finland) was used. Vitamin A and E and beta carotene concentrations in serum were determined using the HPLC system Ultimate 3000 (Dionex, USA). Blood samples for vitamins determination were prepared by extraction (hexane) followed by evaporation and dissolution in mobile phase (methanol).

### Metabolic profile

Serum NEFA and BHB were measured using standardized kits supplied by Randox Laboratories Ltd. The other biochemical parameters such as bilirubin and AST in serum were determined using commercial kits (Biovendor, Czech Republic). An automatic Konelab 20XT biochemical analyzer (Thermo Fisher Scientific, Finland) was used for determination. Serum Ca concentration was analyzed using flame atomic absorption spectrometry - F AAS (SOLAAR, Thermo Scientific, USA). Blood samples for Ca determination were diluted with EDTA 3.

### Statistical analysis

The results obtained were tested for the homogeneity of variances (Hartley-Cochran-Bartlett test) and the normality of distribution (Shapiro-Wilk test). In the event of non-normality, outliers were excluded. The data were analyzed statistically by one-way analysis of variance (ANOVA) followed by the Fisher LSD post-hoc test. All results were expressed as mean value (x) ± standard deviation (SD).

## Results

### Hematological parameters and haptoglobin concentration

Mean values of hematological parameters and haptoglobin concentration in cows with metritis are shown in Table 3. The overall data showed a tendency for lower WBC (6.72 ± 2.19 ×  10^9^/L), lymphocytes (2.19 ± 0.88 × 10^9^/L), monocytes (0.70 ± 0.20 × 10^9^/L), granulocytes (4.00 ± 1.98 × 10^9^/L) and RBC (5.93 ± 1.13 ×  10^12^/L) and higher haptoglobin concentration (1.07 ± 0.57 g/L) in cows with metritis compared to reference values of these parameters.

**Table 3:** Hematological parameters and haptoglobin concentration in cows with metritis (M group)

Variable	M group	Reference values
	(*n* = 21)	
	mean ± SD	
WBC^a^	6.72 ± 2.19	5.00–12.00
Lymphocytes^a^	2.19 ± 0.88	
Monocytes^a^	0.70 ± 0.20	
Granulocytes^a^	4.00 ± 1.98	
RBC^b^	5.93 ± 1.13	5.00–10.00
Haptoglobin (g/l)	1.07 ± 0.57	0.50–0.70

*WBC* white blood cells, *RBC* red blood cells; ^a^ Cell type × 10^9^/l; ^b^ Cell type × 10^12^/l; SD – standard deviation

### Oxidant/antioxidant status

**Fig. 1:**
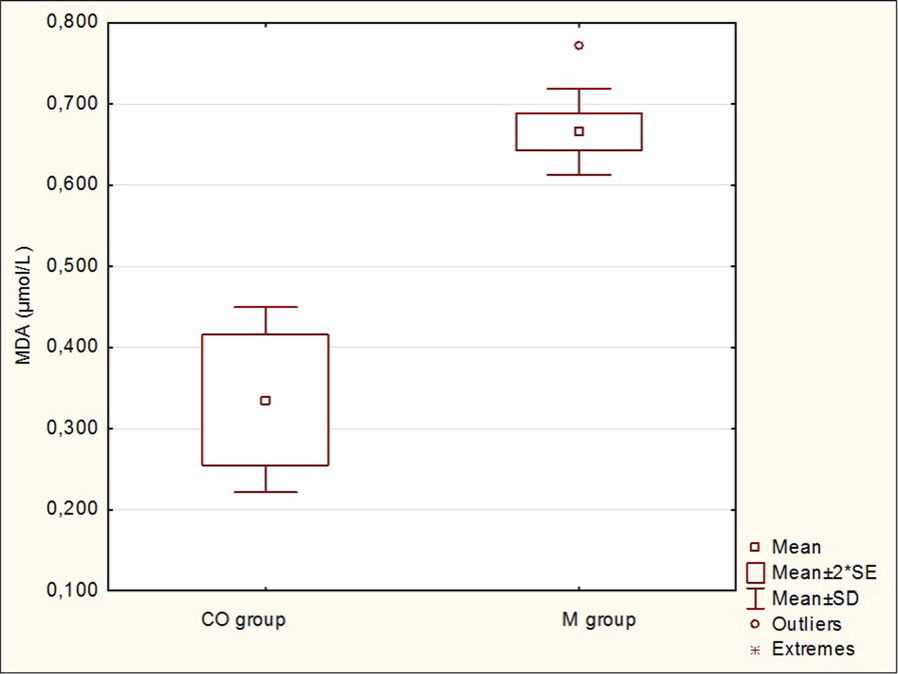
Plasma malondialdehyde (MDA) concentration (μmol/l) in cows with metritis (M group) compared to healthy control cows (CO group). SE – standard error, SD – standard deviation

**Fig. 2:**
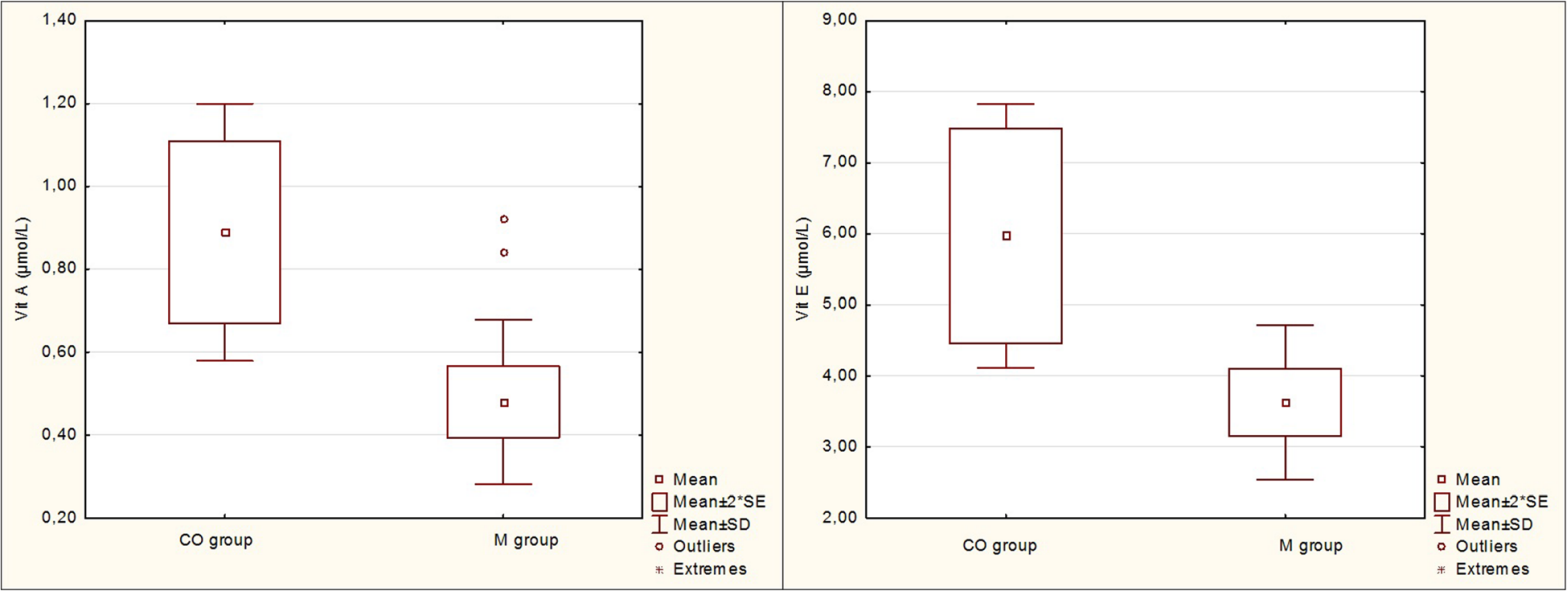
Serum vitamin A and E concentrations (μmol/L) in cows with metritis (M group) compared to healthy control cows (CO group). SE – standard error, SD – standard deviation

As shown in Table 4, significant differences in MDA and vitamin A and E concentrations were observed between the groups. An increased mean plasma MDA concentration was recorded in the group of cows with metritis (0.666 ± 0.053 μmol/l; *P* <  0.001), while vitamin A (0.48 ± 0.20 μmol/l; *P* <  0.01) and vitamin E (3.63 ± 1.09 μmol/l, *P* <  0.01) concentrations were decreased compared to the control group (Figs. 1 and 2). A decreased mean beta-carotene concentration (3.74 ± 1.38 μmol/l) was also found in the metritic group, though this was not, however, significant (*P* >  0.05). In the present study, no significant differences in GPx activity and Se and TAS concentrations were observed in cows with metritis (*P* > 0.05).

**Table 4:** Marker of oxidative stress and antioxidant indicators in cows with metritis (M group) and healthy control cows (CO group)

Variable	CO group	M group	*P* value
	(*n* = 8)	(*n* = 21)	
	mean ± SD	mean ± SD	
MDA (μmol/l)	0.336 ± 0.114	0.666 ± 0.053	< 0.001
GPx (μkat/l)	940.0 ± 194.3	1066.0 ± 134.5	> 0.05
Se (μg/l)	173.4 ± 28.2	184.5 ± 18.0	< 0.05
TAS (mmol/l)	0.92 ± 0.14	1.02 ± 0.06	> 0.05
Vitamin A (μmol/l)	0.89 ± 0.31	0.48 ± 0.20	< 0.01
Vitamin E (μmol/l)	5.97 ± 1.86	3.63 ± 1.09	< 0.01
Beta-carotene (μmol/l)	4.70 ± 2.13	3.74 ± 1.38	> 0.05

*MDA* malondialdehyde, *GPx* glutathione peroxidase, *Se* selenium, *TAS* total antioxidant status, *SD* standard deviation

### Metabolic profile

An increased concentrations of BHB (0.87 ± 0.29 mmol/l; *P* <  0.05), NEFA (0.75 ± 0.39 mmol/l; *P* <  0.05), AST (1.61 ± 0.37 μkat/l; *P* <  0.05) and bilirubin (5.7 ± 1.5 μmol/l; *P* <  0.001) were recorded in cows with metritis, while the Ca concentration was slightly decreased, though this was not significant compared to the healthy cows (*P* > 0.05; Table 5).

**Table 5:** Metabolic profile in cows with metritis (M group) and healthy control cows (CO group)

Variable	CO group	M group	*P* value
	(*n* = 8)	(*n* = 21)	
	mean ± SD	mean ± SD	
BHB (mmol/l)	0.62 ± 0.14	0.87 ± 0.29	< 0.05
NEFA (mmol/l)	0.37 ± 0.19	0.75 ± 0.39	< 0.05
Ca (mmol/l)	2.37 ± 0.25	2.24 ± 0.24	> 0.05
AST (μkat/l)	1.21 ± 0.15	1.61 ± 0.37	< 0.05
Bilirubin (μmol/l)	2.4 ± 0.7	5.7 ± 1.5	< 0.001

*BHB* beta-hydroxybutyrate, *NEFA* non-esterified fatty acids, *AST* aspartate aminotransferase, *SD* standard deviation

### Milk production and composition

**Fig. 3:**
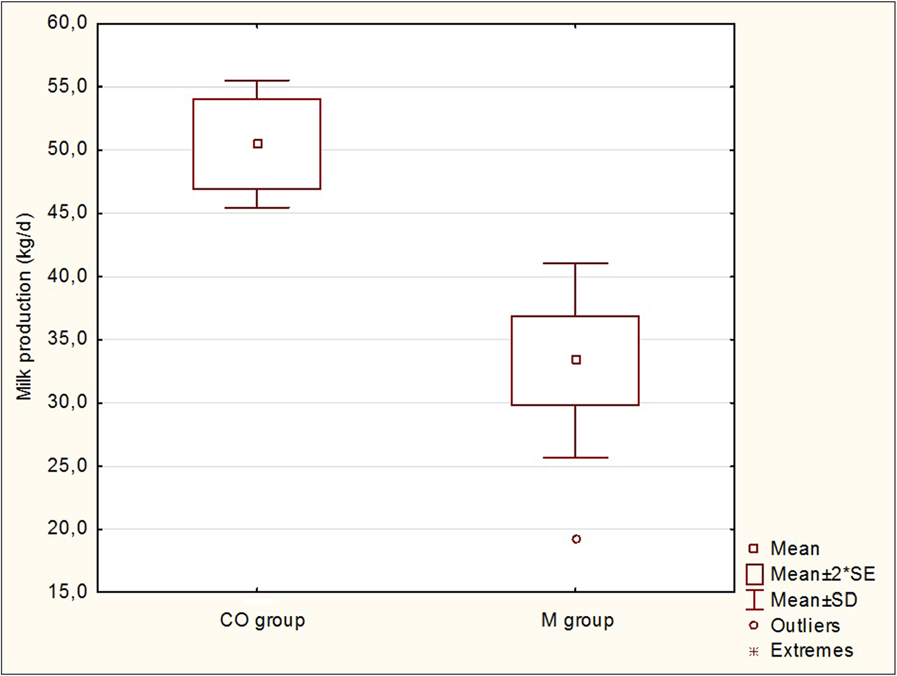
Milk production (kg/d) in cows with metritis (M group) compared to healthy control cows (CO group). SE – standard error, SD – standard deviation

Milk production and values of milk fat, protein, lactose, SCC and milk urea concentration in cows with metritis and in healthy cows in the first month of lactation are shown in Table 6. Milk production was decreased in the cows with metritis as compared to the healthy cows (33.4 ± 7.7 vs 50.5 ± 5.0 kg/d; *P* < 0.001). The difference in milk production between cows with metritis and healthy control cows is shown in Fig. 3. The fat content in milk was increased in metritic cows compared to the control group (3.69 ± 0.56%). SCC showed a tendency to be higher in cows with metritis (97 ± 98 vs 71 ± 80 × 10^3^ cells/ml; *P* > 0.05), whereas protein, lactose and milk urea did not show significant differences between the metritic group and the control group (*P* > 0.05).

**Table 6:** Milk production and milk composition in cows with metritis (M group) and healthy control cows (CO group)

Variable	CO group	M group	*P* value
	(*n* = 8)	(*n* = 21)	
	mean ± SD	mean ± SD	
Milk production (kg/d)	50.5 ± 5.0	33.4 ± 7.7	< 0.001
Fat (%)	3.19 ± 0.53	3.69 ± 0.56	< 0.05
Protein (%)	3.25 ± 0.17	3.19 ± 0.32	> 0.05
Lactose (%)	5.01 ± 0.21	4.95 ± 0.23	> 0.05
SCC (10^3^ cells/ml)	71 ± 80	97 ± 98	> 0.05
Urea (mg/100 ml)	21.8 ± 5.8	23.2 ± 4.4	> 0.05

*SCC* somatic cell count, *SD* standard deviation

## Discussion

The detection of metritis in cows was based on clinical and rectal examination. Rectal examination revealed increased uterine size, thickened uterine wall and increased uterine tone. The main factor for the diagnosis of metritis was the evaluation of discharge of varying color and consistency. Another parameter used to diagnose the disease was an increased rectal temperature. In our study, the rectal temperature in metritic cows ranged from 38.4–39.6 °C (Table 2). Thus, almost half of the experimental cows did not have a fever, according to an established literature definition of fever in metritic cows (> 39.5 °C). However, the cows were included in the experiment due to other clinical signs that confirmed the presence of the disease. Only 3 cases of dystocia were found in metritic cows, a predisposing factor for metritis, but not the only one. Metritis is a multifactorial disease. In addition to hypocalcaemia (which was not found in our study), high levels of NEFA and probably genetic factors also contribute to its development.

### Hematological parameters and haptoglobin concentration

In addition to clinical signs, the determination of hematological parameters and haptoglobin concentration were also used to verify the occurrence of metritis in dairy cows. The total count of WBC is used as a marker of infections and inflammation. A decrease in WBC may occur in the case of bacterial and viral infections such as metritis, mastitis and pneumonia. Leukocytes, particularly neutrophils, are transferred to the damaged tissue or the site of infections within 2 h [27]. In the present study, a decrease in WBC (6.72 ± 2.19 cells × 10^9^/l) was also observed in metritic cows compared to physiological range in cattle [28, 29] (Table 3). Furthermore, the number of lymphocytes (2.19 ± 0.88 cells × 10^9^/l) and monocytes (0.70 ± 0.20 cells × 10^9^/l) was decreased in dairy cows with metritis. The results are in agreement with the previous study by Barragan et al. [30] in which decreased WBC (7.62 ± 0.91 cells × 10^6^/ml), lymphocytes (4.13 ± 0.58 cells × 10^6^/ml) and monocytes (0.10 ± 0.20 cells × 10^6^/ml) were recorded in cows with clinical metritis. The reason for decreased WBC in dairy cows with metritis is apparently the accumulation of leukocytes in the uterine lumen, as already mentioned in previous studies. Barragan et al. [30] reported decreased erythrocytes in cows with clinical metritis (6.85 ± 0.10 cells × 10^6^/μl) compared to cows without clinical metritis as a cause of internal hemorrhage due to rupture of the uterine submucosa blood vessels. The number of erythrocytes was decreased in cows with metritis (5.93 ± 1.13 cells × 10^12^/l) in our study as compared to physiological ranges in cattle [28] (Table 3).

Haptoglobin is an acute phase protein widely used in cattle. It binds free hemoglobin and thereby prevents hemoglobin oxidative activity [31]. Under physiological conditions haptoglobin is present at a low concentration [32] (Table 3) and increases during acute phase response. The release of haptoglobin occurs as a part of the inflammatory response [33]. Several studies have suggested the possibility of using haptoglobin to indicate the occurrence of metritis in cows. Huzzey et al. [34] reported that cows with a haptoglobin concentration of around 1 g/l or more at 3 days after parturition are 6.7 times more likely to be diagnosed with metritis. The studies by Hirvonen et al. [33], Sheldon et al. [35], Chan et al. [36] and Pohl et al. [37] have shown a positive association between haptoglobin concentration and metritis in dairy cows. Barragan et al. [30] recorded an increased haptoglobin concentration in cows with clinical metritis as compared to healthy cows, suggesting that these cows experienced a systematic inflammatory response. A significantly increased haptoglobin concentration in cows with metritis as compared to a control group (0.32 ± 0.07 vs 0.12 ± 0.01 mg/ml) was also observed in the study by Dervishi et al. [38]. An increased haptoglobin concentration in metritic cows (1.07 ± 0.57 g/l) was also found in our study compared to physiological ranges in cattle (Table 3).

### Oxidant/antioxidant status

The main goal of this study was to assess the oxidant/antioxidant balance in dairy cows with metritis compared to healthy controls by measuring indicators of oxidative stress and antioxidant parameters. The periparturient period, or the early postpartum period to be exact, is generally characterized by the depletion of antioxidants and results in an imbalance between pro-oxidants and antioxidants. Production of ROS followed by oxidative stress, which may be one of the reasons for the higher incidence of diseases after parturition, is increased because of the high energy demand caused by rapid differentiation of secretory parenchyma, intense mammary gland growth and the onset of milk synthesis [17, 22].

MDA, a secondary intermediate of lipid peroxidation, is used as a biomarker of lipid peroxidation and oxidative damage [39]. Lipid peroxidation is a major consequence of oxidative stress and leads to oxidative damage. Lipids are oxidized by enzymes and by non-enzymatic oxidants. Polyunsaturated fatty acids (PUFAs), fatty acids with more than one double bond, are easily oxidized by free radical-mediated chain oxidation and are the major substrates of lipid peroxidation [39, 40].

In the present study, the plasma MDA concentration was increased (*P* < 0.001) in cows with metritis, while a decrease in vitamin concentrations (*P* < 0.01) was also observed. The results of the study by Kizil et al. [24], in which an increased mean MDA concentration and significantly decreased vitamin A, E and C and beta-carotene concentrations were also found in cows with puerperal metritis as compared to the control group, suggested an impaired antioxidant system and accelerating peroxidation reactions in cows with metritis. An increased MDA concentration indicates an insufficient amount of antioxidants and, on the other hand, an increase of ROS, including lipid peroxidation products, followed by an increased risk of oxidative stress and the incidence of diseases [20, 41]. The decline in blood concentrations of vitamin A and vitamin E that occurs in dairy cows around calving [42] is caused mainly by an increase in vitamin concentrations in the colostrum [43]. Vitamins (A, E) and the vitamin A precursor beta-carotene, as well as trace minerals (Se) and polyphenols, are non-enzymatic antioxidants [44] and can be supplemented in the diet. According to the study by Pontes et al. [45], supplementation with vitamin E during the prepartal period resulted in a reduced incidence of stillbirth and retained fetal membranes in dairy cows. The study by Michal et al. [46] recorded that beta-carotene supplementation reduces the incidence of metritis in dairy cows. Vitamin A could play a role in resistance to infectious disease in dairy cows during the periparturient period [4, 47].

Kendal and Bone [48] recorded that oxidative stress and its immune consequences are caused not only by a deficiency of vitamins, but also by a lack of trace elements such as selenium in the diet. The selenium status in organisms, especially in the periparturient period, is extremely important to health, immunity and growth. Selenium deficiency in dairy cows in this period contributes to greater depletion of antioxidants and the overall inability of the antioxidant system to function [49]. Selenium sufficiency is essential for GPx which, as a selenium-dependent antioxidant enzyme, is also used in indirect determination of the selenium status and indicates long-term selenium supplementation [49, 50].

An expected decrease of antioxidant activity around calving could result from depletion in the fight against ROS which occur in higher concentrations in this period. O’Boyle et al. [51] suggested that reduced antioxidant potential is more likely due to a depleted antioxidant defense mechanism needed to reduce accumulated concentrations of ROS. In the present study, a decline in GPx activity in cows with metritis compared to healthy cows was not observed. Our results are not in line with the findings of Kizil et al. [24] which found decreased GPx activity in cows with acute puerperal metritis compared to a control group, indicating a decrease in GPx activity in association with lipid peroxidation followed by deterioration in the oxidative stress condition. The reason that changes in GPx activity were not found between the groups is probably a sufficient concentration of selenium in dairy cows. Compared to reference values in cattle, in which the whole-blood Se concentration regarded as a reference value is 100/130 μg/l [49, 50], the Se concentrations were increased in both groups (Co group – 173.4 ± 28.2 μg/l, M group – 184.5 ± 18.0 μg/l). Based on this fact, we can state that the selenium concentration differences between the metritic and control groups are not so prominent due to the high selenium supplementation in dairy cows. There is also no significant decrease in GPx activity in the postpartum period, neither in healthy cows nor in the cows with postpartum diseases such as metritis.

**Fig. 4:**
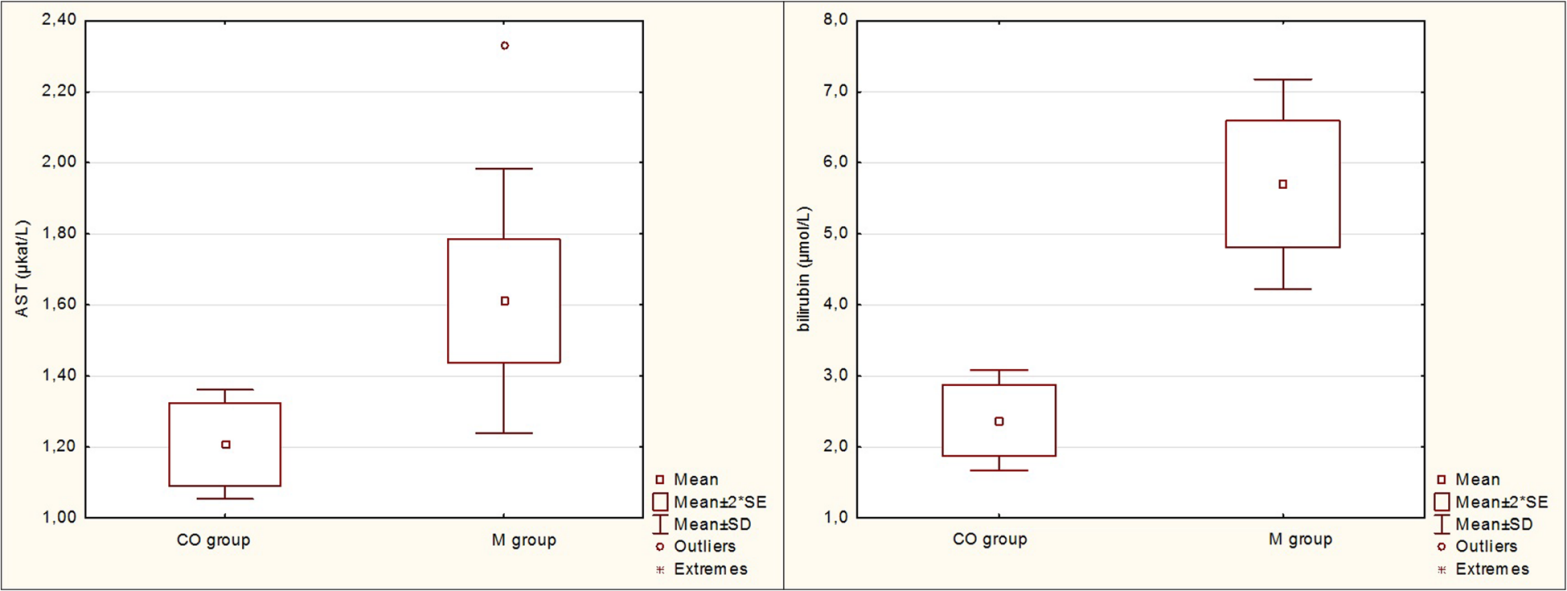
Serum aspartate aminotransferase (AST) activity (μkat/l) and bilirubin concentration (μmol/l) in cows with metritis (M group) compared to healthy control cows (CO group). SE – standard error, SD – standard deviation

According to Ghiselli et al. [52], the TAS evaluates the overall antioxidant capacity in the organism and can therefore provide information about antioxidants in serum. Bionaz et al. [53] reported that a decreased concentration of vitamins A and E and TAS can support a diagnosis of retained placenta in cows, in addition to decreased Ca concentration and increased concentrations of NEFA and BHB. Based on these findings and the results of the present study, in particular the concentration of vitamins, we also expected a decreased TAS concentration in dairy cows with metritis as compared to the control group. However, no significant differences in TAS concentrations in cows were found between the groups (1.02 ± 0.06 vs 0.92 ± 0.14 mmol/l; *P* > 0.05). This could be explained by the fact that although the concentration of vitamins was reduced in metritic cows, other parameters with an antioxidant effect, such as for example the bilirubin concentration, may be increased (Fig. 4, Table 5) and affect TAS as an indicator of total amount of antioxidants in organism.

### Metabolic profile

**Fig. 5:**
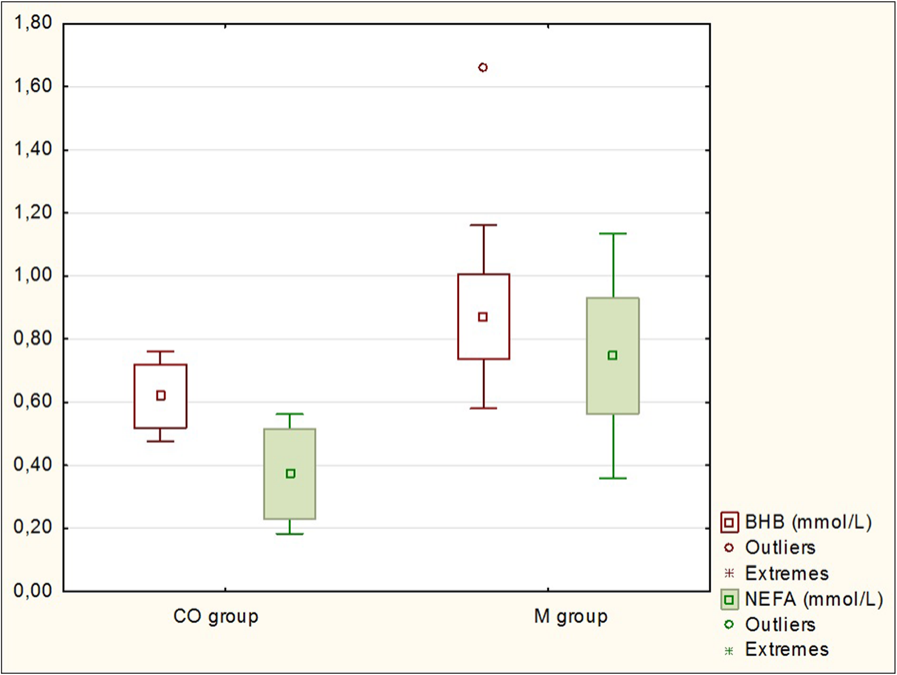
Serum non-esterified fatty acids (NEFA) and beta-hydroxybutyrate (BHB) concentrations (mmol/l) in cows with metritis (M group) compared to healthy control cows (CO group)

Furthermore, metabolic profiles including NEFA, BHB, Ca, bilirubin concentration and AST activity have been used in an attempt to monitor metritis in dairy cows more closely. Insufficient energy intake in the periparturient period in dairy cows results in an increase in NEFA concentrations as a response to lipomobilization [54]. Lower feed intake is associated with the incidence of metritis in dairy cows which have decreased appetite and therefore spend less time feeding [55, 56]. Determination of serum NEFA concentration is considered a marker of energy balance [57]. Hammon et al. [58] suggested that cows experiencing NEB around parturition are more susceptible to periparturient immunosuppression. Significantly increased blood NEFA and BHB concentrations and lower dry matter intake in cows with metritis were reported in the study. Similar findings were observed in the present study (Fig. 5), where also a significantly increased NEFA (0.75 ± 0.39 mmol/l) and BHB (0.87 ± 0.29 mmol/l) concentrations in metritic cows were found. In the study by Dervishi et al. [38] no significant differences in NEFA and BHB concentrations were observed in dairy cows with metritis compared to healthy cows. Similarly, Barragan et al. [30] did not find any changes in BHB concentration between the metritic and control group of cows. Barragan et al. [30] also reported that the body condition score (BCS) in cows with clinical metritis was lower compared to healthy cows, partly due to lower feed consumption in the late prepartum period. In the present study was also observed decreased BCS value in cows with metritis compared to control group (3.08 ± 0.16 vs 3.38 ± 0.26; *P* < 0.01). However, additional information about feed intake in dairy cows would be required to suggest a hypothesis.

Ingvartsen and Moyes [59] and Martinez et al. [60] recorded a link between hypocalcemia and clinical metritis. These studies found that metabolic diseases such as hypocalcemia impair the immune system function in dairy cows, for which reason the incidence of infectious diseases increases in early postpartum. In the present study, the Ca concentration did not differ between the metritic group and the control group of cows (2.24 ± 0.24 vs 2.37 ± 0.25 mmol/l). In contrast, the study by Barragan et al. [30] found a decreased Ca concentration in cows with clinical metritis as compared to healthy cows. This may be because in addition to the decreased concentration of calcium predisposing the occurrence of metritis, there are also several other factors such as the number and type of bacteria, the immune response of postpartum cows, endometrial regeneration and endocrine factors which influence the development of metritis.

The increase in serum AST concentrations after calving may be associated with increasing synthetic activity in the liver due to protein mobilization [61] or with slight liver damage caused by lipomobilization [54]. LeBlanc et al. [4] reported that liver function during NEB in the periparturient period is one of the predisposing factors for mastitis in early lactation. This could explain the findings in our study in which increased AST activity (*P* < 0.05) and bilirubin concentration (*P* < 0.001) were reported in cows with metritis compared to healthy cows. The study by Moretti et al. [62] found that hypocalcemia and increased AST and GMT activity can also often be observed in cows with a retained placenta compared to healthy controls.

### Milk production and composition

Metritis and uterine diseases in general are associated with decreased milk yield [11, 38, 63]. Mordak and Anthony [64] reported a link between reduced milk yield, lower BCS, decreased fertility and cows with a retained placenta. Our results are in agreement with the study by Dervishi et al. [38] in which milk production was significantly decreased in cows with metritis (35.12 ± 2.10 vs 43.01 ± 1.62 kg/d). The results in the present study also showed changes in milk composition in metritic cows. The differences in the fat content of milk between the metritic and healthy cows (3.69 ± 0.56 vs 3.19 ± 0.53%; *P* < 0.05) are associated with milk production in individual groups. Milk fat content is known to decrease with increasing milk yield and in the first half of lactation. The lower fat content of milk in the control group may be due to the high milk production in these cows (almost double that of cows with metritis). On the other hand, increased fat content of milk in early lactation is associated with the development of energy deficiency and subsequent occurrence of ketosis and lipomobilization syndrome. Lipolysis occurs during NEB and fatty acids used for the synthesis of milk fat are released from the fat reserves of dairy cows [65], which may partly explain significantly increased fat content of milk in metritic cows. The increased SCC in the milk of metritic cows compared to healthy cows was also observed in the study by Dervishi et al. [38], in which a tendency for increase was found (91.75 ± 21.19 vs 28.33 ± 5.63 × 10^3^ cells/ml; *P* = 0.05).

## Conclusions

In cows with post-partum metritis, an elevated marker of oxidative stress and conversely a decline in antioxidant concentrations were observed compared to healthy cows. The metabolic profile, including energy balance indicators and liver markers, also demonstrated significant differences between the groups. The results of the study showed a relation between oxidative stress and metritis. This suggests that cows with metritis in early postpartum are exposed to a higher degree of oxidative processes. Our findings also showed that the incidence of metritis negatively affected milk production and composition in dairy cows. It can be concluded that monitoring the energy balance (BCS, NEFA), the total mixed ration composition and metabolic imbalances followed by possible preventive action in the prepartal period is important and can contribute to prevent the incidence of such diseases and possible negative consequences in dairy cows. For further research, it would be advisable to follow the developmental line from the prepartal to the postpartum period in cows with metritis compared to a control group.

## Data Availability

The datasets used and/or analysed during the current study are available from the corresponding author on reasonable request.
